# Interactions between Pb(II) and Cassava Residue Biochar: Adsorption, Complexation, and In Situ Application

**DOI:** 10.1021/acsomega.6c01742

**Published:** 2026-04-29

**Authors:** Ricardo Rafaell da Silva, Andrea Pires Fernandes, Sivaldo Soares Paulino, Luciana Camargo de Oliveira, André Gustavo Ribeiro Mendonça, Wander Gustavo Botero

**Affiliations:** † Graduate Program in Chemistry and Biotechnology, Institute of Chemistry and Biotechnology, Federal University of Alagoas (UFAL), Maceió, AL 57072-900, Brazil; ‡ Department of Physics, Chemistry and Mathematics, Federal University of São Carlos (UFSCar), Rodovia João Leme dos Santos, SP-264, Km 110, Sorocaba, São Paulo 18052-780, Brazil

## Abstract

The production of biochar through slow pyrolysis under controlled conditions represents a promising strategy for converting agro-industrial residues into functional materials for environmental remediation. This approach contributes to sustainability by providing an alternative to the disposal of waste with no specific application. In this context, the present study aimed to evaluate the interaction of cassava peel-derived biochars, produced at different pyrolysis temperatures (350 °C, 450 °C, 500 °C, and 550 °C), with lead (PbII) ions and to investigate their efficiency in removing this contaminant under environmentally relevant conditions. The biochars were characterized by elemental analysis. To assess their performance in Pb­(II) removal, adsorption isotherms, equilibrium time, complexation capacity, and in situ application tests were conducted. Elemental analysis revealed a clear influence of the pyrolysis temperature on the physicochemical properties of the biochars. Among the adsorption isotherm models tested, the Langmuir model provided the best fit to the experimental data, indicating that Pb­(II) adsorption occurs on a homogeneous monolayer surface without interactions between adsorbed ions. The maximum adsorption capacity for Pb­(II) reached 37.27 mg g^–1^, a relatively high value compared to similar studies, and was obtained with biochar produced at the highest pyrolysis temperature (550 °C). The complexation capacity ranged from 37.60 to 39.11 mg g^–1^. In the in situ application, the biochar produced at 550 °C using 200 mg exhibited the highest Pb­(II) retention (15.56 mg g^–1^). Overall, cassava peel-derived biochars demonstrate considerable potential as low-cost, environmentally friendly materials for mitigating Pb­(II) contamination in aquatic environments.

## Graphical abstract

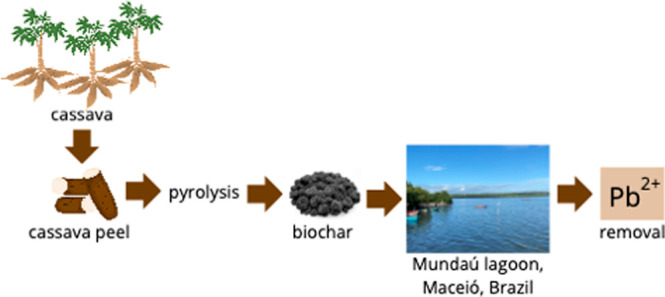

## 1 Introduction

Water contamination by potentially toxic metals (PTMs) is a major global concern due to its severe impacts on human health. These chemical species are highly resistant to biodegradation and therefore pose significant threats to food security and the environment.^1^ Anthropogenic activities are the primary sources of PTM contamination, particularly through agro-industrial processes and industrial discharges.

Among the PTM commonly found in the environment, lead (Pb II) is of particular concern because of its widespread distribution and its potential to cause serious health problems in humans.^2^ The World Health Organization (WHO) lists lead among the ten chemicals of greatest public health concern, and the Institute for Health Metrics and Evaluation (IHME) estimated that approximately 1.5 million deaths worldwide in 2021 were attributable to lead exposure. Currently, lead is recognized as one of the most critical environmental pollutants.^3^

Therefore, the removal of Pb­(II) ions using green, efficient, and low-cost technologies is of great importance.^4^ In recent years, Pb­(II) adsorption by solid adsorbents has attracted increasing attention.^4,5^ In this context, biochar (BC), produced by pyrolysis, has emerged as a promising strategy for the removal of PTM from water.^6–8^

Biochar is defined as the carbonaceous material obtained from the thermal decomposition of organic biomass under oxygen-limited conditions.^9^ It is primarily composed of carbon, exhibits fine granularity, and is highly resistant to decomposition.^9–11^ In addition, biochar has been widely discussed as a sustainable alternative for the valorization of agro-industrial residues^9,10^ and as a material with strong potential for environmental remediation.^12^

BC produced under low-temperature pyrolysis generally exhibit a disorganized carbonaceous molecular structure, consisting of flat layers of randomly linked aromatic rings, along with an inorganic fraction derived from metals present in the feedstock.^10^ The surface chemistry of BC may vary, displaying hydrophobic, hydrophilic, basic, and/or acidic properties, which contribute to their reactivity.^9^ Owing to these characteristics, BCs have been widely studied as adsorbents for the remediation of natural and wastewater systems.^7,8^ The presence of functional groups such as hydroxyl, carboxyl, and phenolic groups on the BC surface plays a crucial role in their interactions with PTM, including Cd­(II) and Pb­(II) ions.^13,14^

The immobilization of Pb­(II) by biochar is governed by multiple mechanisms acting simultaneously rather than a single dominant process. Among these, surface complexation plays a key role, involving the interaction of Pb^2+^ ions with oxygen-containing functional groups (e.g., carboxyl, hydroxyl, and phenolic groups) present on the biochar surface. In addition, ion exchange mechanisms may occur, particularly through the replacement of exchangeable cations such as Ca^2+^, Mg^2+^, and K^+^ associated with the mineral fraction of the biochar. Electrostatic attraction also contributes to Pb­(II) removal, especially when the solution pH exceeds the point of zero charge (pHpzc), resulting in a negatively charged surface that favors the uptake of cationic species. Furthermore, precipitation processes can significantly influence Pb immobilization, particularly at higher pH values or in biochars rich in inorganic components, leading to the formation of low-solubility phases, such as Pb­(OH)_2_, PbCO_3_, or Pb-phosphates. The relative contribution of each mechanism depends on factors such as pyrolysis temperature, surface chemistry, mineral composition, and solution conditions.^7,9,10^

Nevertheless, further research is needed to elucidate the mechanisms underlying BC-contaminant interactions, particularly regarding complexation capacity (CC), adsorption performance, and most importantly, their effectiveness under environmentally relevant conditions.

Therefore, the present study aimed to investigate the interaction of biochars derived from cassava peel (CP-BC), produced at different pyrolysis temperatures (350 °C, 450 °C, 500 °C, and 550 °C), with Pb­(II) and evaluate their potential for Pb­(II) remediation in a real environmental system. The use of biochars derived from cassava peel is relevant for agro-industrial contexts, and the use of techniques such as AGNES and TF-UF is innovative in environmental studies. Furthermore, in situ application contributes to a realistic understanding of the use of biochar in environmental remediation.

## 2 Materials and Methods

### 2.1 Reagents and Solutions

All reagents used were of analytical grade and acquired through Merck (Darmstadt, Germany) and Sigma-Aldrich (St. Louis, USA). All solutions were prepared with ultrapure water obtained from a Gehaka system (São Paulo, Brazil) with 18.2 MΩ•cm (at 25 °C). Before use, all working materials were previously washed with 10% (v/v) HNO_3_ and ultrapure water.

### 2.2 Production and Characterization of Cassava Peel Biochar

The BCs used in this study were produced and previously characterized by Silva et al.^15^ They were obtained through pyrolysis of cassava peel (CP) samples in a laboratory-scale unit equipped with a 1 kW JUNG LT6 tubular furnace (Blumenau, Brazil) electrically heated with a maximum operating temperature of 1000 °C and a cooling system for liquid-phase collection. The experiments were conducted at a fixed heating rate of 10 °C min^–1^ with a residence time of 90 min. Pyrolysis was performed at four temperatures: 350 °C (BC1), 450 °C (BC2), 500 °C (BC3), and 550 °C (BC4). After production, the BCs were cooled to room temperature (25 ± 1 °C), ground using a porcelain mortar and pestle, and sieved through a 42-mesh sieve (<355 μm).^15^

According to Silva et al.,^15^ the physicochemical properties of the BC were investigated by thermogravimetric analysis (TGA) using a Shimadzu DTG-60TG (Tokyo, Japan), Fourier-transform infrared spectroscopy (FTIR) with a Varian 660-IR spectrometer (Berlin, Germany), and X-ray diffraction (XRD) with a Shimadzu XRD-6100 diffractometer (Tokyo, Japan). In addition, the concentrations of metallic species (Al, Ba, Cd, Co, Cr, Cu, Mn, Mo, Ni, Pb, Sr, and Zn), pH values, and point of zero charge (pHpzc) were determined. In the present study, complementary elemental analysis of BC was also performed.

Elemental composition (C, H, N, and O) was determined by using a Thermo Finnigan Flash EA 1112 elemental analyzer. This allowed quantification of the carbon, hydrogen, and nitrogen contents in both the raw material and BC produced at different pyrolysis temperatures. The percentage values of C, H, and N were converted into atomic ratios by dividing each percentage by the corresponding atomic mass. Oxygen content (O %) was calculated by difference according to the equation:

O%=100−(C%+H%+N%)

Subsequently, the atomic ratios H/C, O/C, and N/C were calculated.^16^

### 2.3 Adsorption Experiments

Adsorption isotherm experiments were conducted in triplicate at pH 6.0, a condition of environmental relevance and optimal for adsorption kinetics.^15^ BC samples were tested for their interaction with Pb­(II) ions under agitation at 100 rpm and 25 ± 1 °C for 24 h by using an orbital shaker. This time (24 h) was chosen based on preliminary kinetic tests, which indicated that equilibrium conditions were reached within this time frame. For each assay, 50 mg of BC was weighed and transferred to conical tubes, to which 15 mL of Pb­(II) solution was added at concentrations of 5.0, 10.0, 20.0, 30.0, 40.0, 50.0, 75.0, 100.0, 150.0, and 200.0 mg L^–1^.

The equilibrium adsorption data for Pb­(II) were analyzed using the Langmuir and Freundlich isotherm models to establish the relationship between the amount of adsorbate and the equilibrium concentration at pH 6.0. The initial pH of the solutions was adjusted to 6.0 using diluted solutions of HCl and/or NaOH, and MES buffer ((2-(*N*-morpholino)­ethanesulfonic acid)) was used. Furthermore, the pH was monitored throughout the experiments to ensure that no significant variations occurred during the contact time. Control (blank) experiments were performed under identical experimental conditions in the absence of biochar to assess potential Pb­(II) removal not associated with the adsorbent.

According to the Langmuir model, the adsorption capacity (*q*_e_) is given by eq 1:

1 $$q_{e}=\frac{q_{\max}K_{L}C_{e}}{1+K_{L}C_{e}}$$

where *q*_e_ is the equilibrium adsorption capacity (mg g^–1^), *q*_max_ is the maximum adsorption capacity (mg g^–1^), *K*_L_ is the Langmuir constant (L mg^–1^), and *C*_e_ is the equilibrium concentration of Pb­(II) in solution (mg L^–1^).

This equation can be expressed in its linearized form, as shown in eq 2:

2 $$\frac{C_{e}}{q_{e}}=\frac{1}{q_{\max}}C_{e}+\frac{1}{K_{L}q_{\max}}$$

The degree of feasibility and spontaneity of the adsorption reaction can be assessed by evaluating the equilibrium parameter or separation factor (*R*_L_) (eq 3). This dimensionless parameter indicates whether the adsorption process is favorable or unfavorable.

3 $$R_{L}=\frac{1}{1+K_{L}C}$$

where (*C*) is the highest initial concentration (mg L^–1^) and (*K*_L_) is the Langmuir constant. Values of (*R*_L_ > 1) indicate an unfavorable process, (*R*_L_ = 1) indicates a linear isotherm, (0 < *R*_L_ < 1) indicates favorable adsorption, and (*R*_L_ = 0) corresponds to an irreversible process.^17^

The Freundlich isotherm is an empirical model that assumes adsorption occurs in multilayers on heterogeneous surfaces.^18^ The Freundlich model is described by eq 4, with its linearized form given by eq 5:

4 $$q_{e}=K_{F}C_{e}^{1/n}$$

5 $${logq}_{e}={logK}_{F}+\frac{1}{n}{logC}_{e}$$

where (*q*_e_) is the amount of solute adsorbed at equilibrium (mg g^–1^), (*C*_e_) is the equilibrium concentration of the adsorbate (mg L^–1^), and (*K*_F_) and (*n*) are the Freundlich constants related to adsorption capacity and adsorption intensity, respectively. The modeling was done by using Origin software.

In addition to *R* ^2^ and χ^2^, model selection was further evaluated using the Akaike Information Criterion (AIC) and Bayesian Information Criterion (BIC), calculated according to:

AIC=nln(RSS/n)+2kandBIC=nln(RSS/n)+kln(n)

where *n* is the number of experimental data points, RSS is the residual sum of squares, and k is the number of model parameters.

### 2.4 Equilibrium Time and Complexation Capacity

The equilibrium and complexation capacity (CC) experiments were conducted using a tangential flow ultrafiltration system (TF-UF).^19^ This technique is versatile and user-friendly as it does not require the addition of reagents, thereby preventing potential alterations to the samples. The system was equipped with a 1 kDa, 47 mm diameter membrane (poly­(ether sulfone), Gelman Pall-Filtron OMEGA/regenerated cellulose), which permits only the passage of free Pb­(II). The concentration of free metal species in the equilibrium and CC experiments was determined by electrochemical chronopotentiometric stripping in Nernstian equilibrium under conditions free of concentration gradients (AGNES).

#### 2.4.1 Complexation Equilibrium

The equilibrium experiment was conducted to determine the minimum time required for Pb (II) ions to form complexes with BC. A mass of 10 mg of BC was weighed and dispersed in 450 mL of ultrapure water, yielding a concentration of 22.22 mg L^–1^ BC in the solution. Subsequently, 50 mL of a standard Pb­(II) solution (1000 mg L^–1^) was added, resulting in a final concentration of 100 mg L^–1^ for Pb (II) and 20 mg L^–1^ for BC. To monitor equilibration, ten aliquots of approximately 2.0 mL each were collected at 5, 10, 20, 30, 40, 50, 60, 70, 80, and 90 min. Equilibrium time was determined by plotting the concentration of free Pb­(II) as a function of time.^20^

#### 2.4.2 Complexation Capacity

The complexation capacity (CC) of BC produced under different pyrolysis temperatures (BC1 at 350 °C, BC2 at 450 °C, BC3 at 500 °C, and BC4 at 550 °C for Pb (II) ions) was determined by TF-UF. Masses of 10 mg of each BC were dispersed in water to obtain solutions with a concentration of 20 mg L^–1^. The BC exhibited low solubility, and all experiments were conducted at pH 6.0. Prior to the addition of Pb (II) solution, the system was conditioned by circulating the initial solution for 5 min^20^

After the TF-UF system was adjusted, a 2.0 mL aliquot was collected by filtration at time zero (blank) before Pb­(II) addition. Subsequently, volumes of the Pb­(II) stock solution were added to the BC1–BC4 suspensions to obtain final concentrations of 0.10, 0.20, 0.30, 0.50, 1.00, 2.00, 5.00, 10.00, 25.00, and 50.00 mg L^–1^. After each Pb­(II) addition, the suspensions were stirred for 10 min, filtered, and analyzed for the Pb­(II) concentration using electrochemical chronopotentiometric stripping in Nernstian equilibrium under conditions free of concentration gradients (AGNES).

The CC was determined by plotting the total Pb­(II) concentration against the free Pb­(II) concentration. The CC value corresponded to the intersection point of the two linear segments of the curve.^21^

### 2.5 In Situ Application of Biochars for Lead Retention

The in situ studies were conducted in the Munda-Manguaba Estuarine Lagoon Complex (MMELC), located in the municipality of Maceió, Alagoas, Brazil. The MMELC is a highly productive ecosystem of great importance for the survival of local populations, who rely on its resources for a significant part of their livelihood.^22^

For the in situ experiments, 200, 300, and 500 mg of BC produced under different pyrolysis temperatures—BC1 (350 °C), BC2 (450 °C), BC3 (500 °C), and BC4 (550 °C)—were applied in permeable and sterilized bags at a depth of 40 cm in the water column. The contact time varied from 30 to 480 min at a specific site within the lagoon, selected due to its previously reported high metal concentrations.^23^ All experiments were performed in triplicate, and the elution of lead adsorbed on the biochar was done using 25.0 mL of 1 mol L^–1^ HNO_3_ solution under stirring for 20 min^23^ The determination of other ions present in the lagoon waters was performed by atomic absorption spectrometry (AAS).

### 2.6 Determination of Pb­(II) by AGNES

The free metal ion concentration Pb­(II) was quantified by using the electroanalytical technique absence of gradients and Nernstian equilibrium stripping (AGNES). An Ecochemie μAutolab III and a PGStat 12 were used in conjunction with a Metrohm 663 VA stand (Metrohm, Switzerland). The setup was controlled by GPES 4.9 software from EcoChemie, The Netherlands. A three-electrode configuration was used comprising a Hg thin film plated onto a rotating glassy carbon (GC) disk (2 mm diameter, Metrohm) as a working electrode, a GC rod counter electrode, and a Ag/AgCl reference electrode from World Precision Instruments DRIREF-5 (electrolyte leakage <8 × 10^–4^ μL h^–1^). A Denver Instrument (model 15) and a Radiometer analytical combination pH electrode, calibrated with Titrisol buffers (Merck, Darmstadt, Germany) were used to measure the pH of the samples. The electrochemical measurements were carried out at 25 °C. The calibration was performed at concentrations of 1 to 10 × 10^–8^ mol L^–1^ after measuring the analytical blank in a buffered system with MES. The limits of detection and quantification were 5.5 nmol L^–1^ and 0.035 μmol L^–1^, respectively. All measurements were performed in triplicate and no systematic variation was observed indicating that the chemical equilibrium was achieved in solution.^24,25^

## 3 Results and Discussion

### 3.1 Production and Characterization of Biochar Derived from Cassava Peel

BCs are promising materials for the remediation of rivers and lakes contaminated by PTM. However, their effectiveness strongly depends on their intrinsic properties and interactions with the environment. These properties are influenced by the type and heterogeneity of the raw material, and, most importantly, by the pyrolysis conditions.^26^

Characterization studies are essential to evaluating BC properties. The BCs used in this research were previously characterized by Silva et al.,^15^ who reported significant structural differences resulting from the influence of pyrolysis temperature. While graphitic structures are typically obtained at very high temperatures (≈3500 °C), the BC investigated in this study exhibited organized, condensed aromatic domains without the presence of crystalline graphite.^15^

Silva et al.^15^ report that biochar derived from cassava waste exhibited physicochemical characteristics strongly dependent on pyrolysis temperature, with a progressive increase in aromaticity and degree of carbonization at higher temperatures. Elemental analysis shows a reduction in H/C and O/C ratios, indicating greater structural condensation and a lower presence of oxygenated functional groups. Techniques such as FTIR reveal a decrease in polar surface groups (such as hydroxyls and carboxyls), while XRD suggests the predominance of amorphous structures with possible associated mineral phases. Furthermore, thermal analysis (TGA) demonstrated greater thermal stability of the biochars produced at higher temperatures. Together, these characteristics directly influence adsorptive performance, affecting both the ability to interact with metal ions and the mechanisms involved in the immobilization process.

In addition, elemental analysis was performed to complement the evaluation of the structural characteristics of BC used in this study.

### 3.2 Elemental Analysis

Elemental analysis plays a key role in the characterization of BC and in assessing the degree of degradation after thermal processing. The elemental contents and derived atomic ratios provide essential information about the structural characteristics of BC.

The elemental composition of BC obtained from CP at carbonization temperatures of 350, 450, 500, and 550 °C revealed variations in the percentages of C, H, N, and O as a function of the temperature (Figure 1).

**Figure 1:**
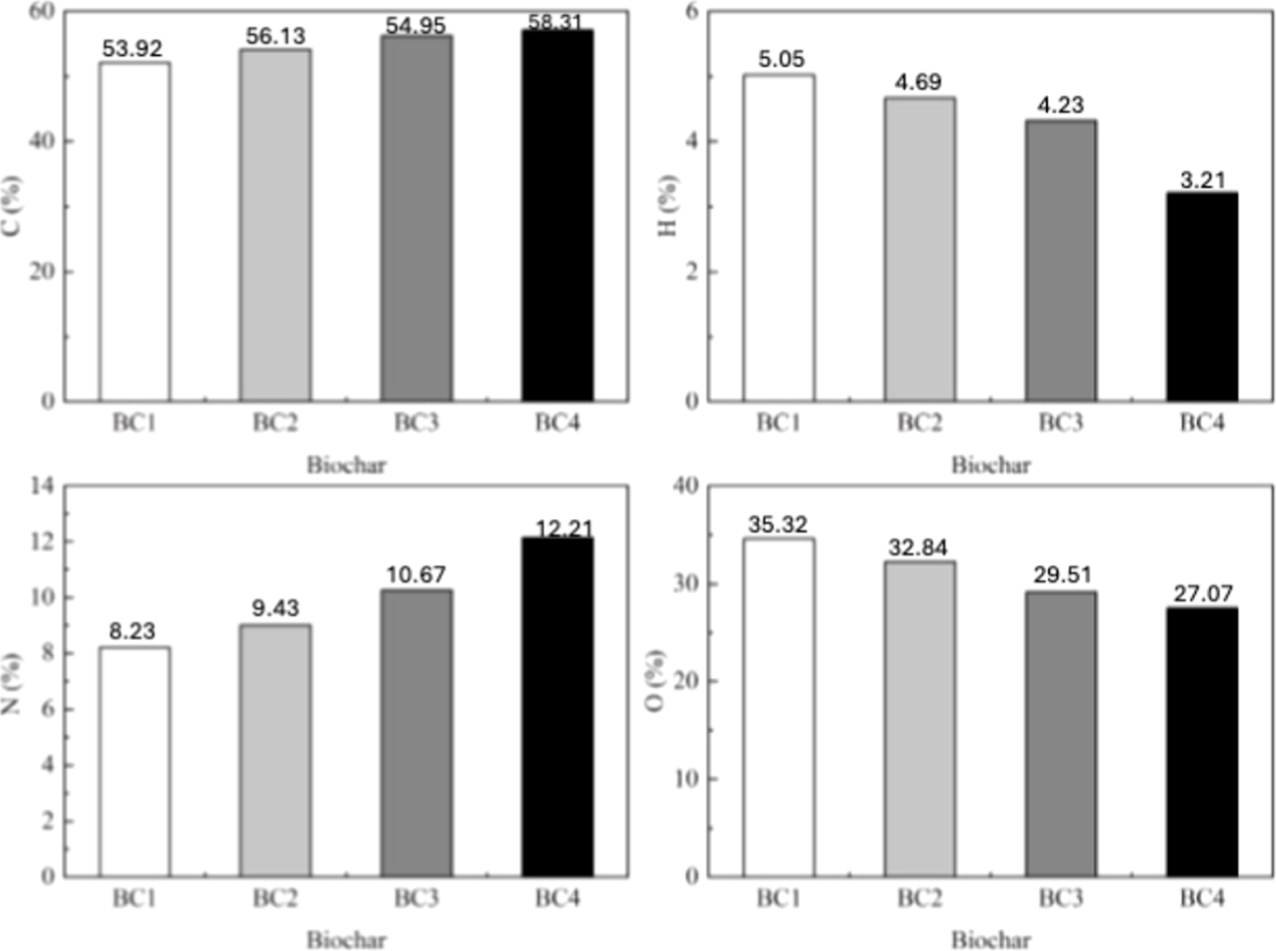
Elemental compositions of biochar (BC) from cassava peel (CP) produced under different temperature conditions: BC1, 350 °C; BC2, 450 °C; BC3, 500 °C; and BC4, 550 °C.

In general, an increase in carbon content and a progressive reduction in hydrogen and oxygen content are observed, while nitrogen shows more variable behavior, but with a tendency toward relative concentration. The increase in the carbon content (from ∼53.92% in BC1 to ∼58.31% in BC4) is directly related to the progressive carbonization of the biomass. During pyrolysis, the degradation of labile components such as cellulose and hemicellulose occurs, releasing volatile compounds (CO_2_, CO, CH_4_), resulting in a solid matrix richer in condensed aromatic carbon. This enrichment indicates a higher degree of aromaticity and structural stability of the biochar.^27^

The H and O contents decreased with increasing pyrolysis temperature, ranging from 6.97 to 36.06% and 7.02 to 20.53%, respectively. This reduction is attributed to the thermal degradation of volatile components, dehydration of organic compounds, and cleavage of weak bonds in the CP structure.^27,28^ The decrease in oxygen content also indicates the degradation of carboxylic functional groups and the formation of more stable aromatic ring structures, as evidenced by the increase in C content.^28,29^ In parallel, the decrease in H content suggests an enrichment in unsaturated carbons (CH_2_–CH_2_).^[^ ^22^ ^]^

Regarding nitrogen, a relative increase is observed (from ∼8.23% to ∼12.21%), which does not necessarily indicate N incorporation but rather a concentration effect due to the loss of other elements (mainly O and H). Part of the nitrogen can be transformed into more stable heterocyclic structures (such as pyridines and pyrroles), contributing to specific properties of biochar such as surface basicity and complexation potential with metals.

These elementary changes reflect important shifts in the structure and functionality of the biochar. The Van Krevelen diagram is widely used to illustrate changes in the elemental composition of BC with an increasing pyrolysis temperature. In this study, higher temperatures resulted in decreased molar ratios of H/C, O/C, and N/C (Figure 2).

**Figure 2:**
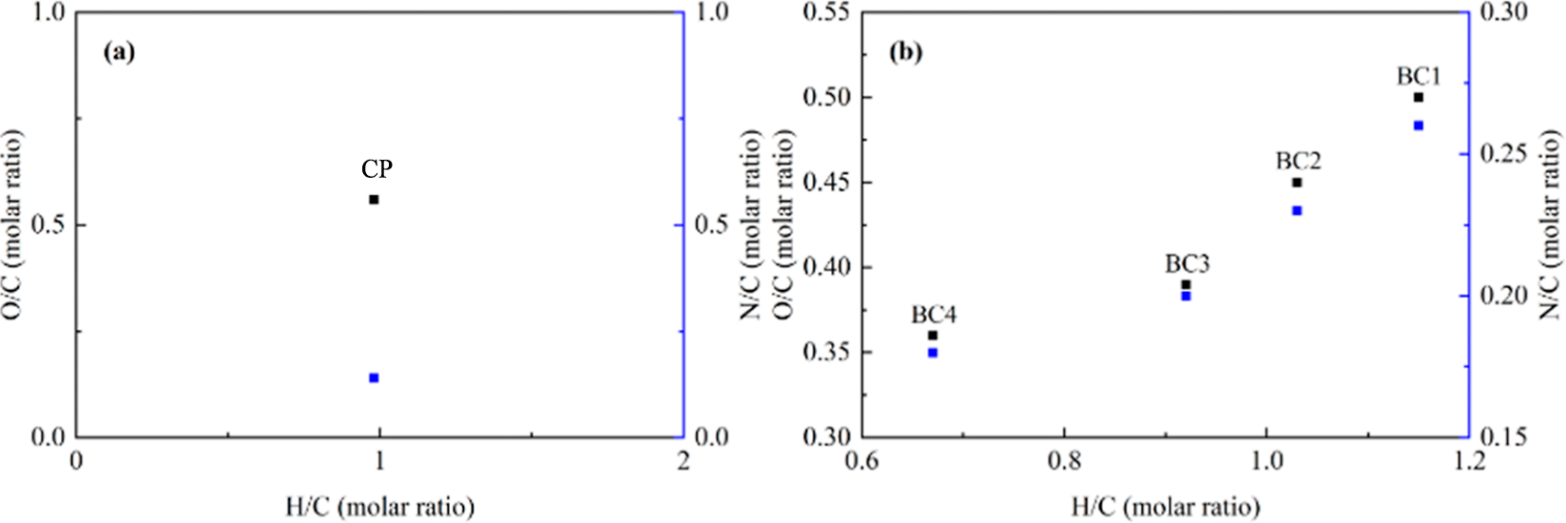
Van Krevelen plot of elemental ratios for (a) cassava peel (CP) and (b) biochars (BCs) produced under different temperature conditions: BC1, 350 °C; BC2, 450 °C; BC3, 500 °C; and BC4, 550 °C.

The reduction in the O/C and N/C ratios with increasing temperature may be attributed to enhanced carbonization, indicating a lower abundance of oxygenated and nitrogen-containing functional groups. A decrease in these groups corresponds to reduced reactivity compared to BC with higher O/C and N/C ratios. The H/C ratio, in turn, is associated with the degree of aromaticity: the lower the ratio, the greater the aromaticity.^21^

Both H/C and O/C ratios reflect the aromaticity and polarity of BC. Accordingly, the observed decreases in these ratios with increasing pyrolysis temperature indicate greater aromaticity and reduced polarity.^21,28^ The degree of aromaticity is critical for BC applications as a soil conditioner since the presence of aromatic carbon reduces susceptibility to decomposition, thereby contributing to climate change mitigation, soil structural improvement, and agricultural productivity.

Thus, BC4, produced at the highest temperature (550 °C), exhibits the greatest stability, making it particularly suitable for agricultural and environmental applications. The trends in elemental composition and atomic ratios with increasing pyrolysis temperature observed in this study are consistent with findings reported in the literature.^28,30,31^

Silva et al.^15^ determined the pHzpc of cassava peel biochar, obtaining results between 6.09 and 7.26, highlighting its use at pH 6 (used in this work).

### 3.3 Adsorption Isotherms

Isothermal modeling enables the evaluation of the theoretical adsorption mechanism best suited to the experimental data, distinguishing between monolayer and multilayer adsorption. In addition, adsorption isotherm models provide key parameters of the adsorption process such as the maximum adsorption capacity of B from CP produced at different pyrolysis temperatures. While the Langmuir and Freundlich models are the most applied, other models can also be employed to gain further insight into the adsorption process.

The Langmuir model assumes that adsorption occurs on a homogeneous monolayer surface without interactions between adsorbed ions, with all surface sites having identical adsorption energies. Under this model, adsorption continues until all of the available sites are occupied. In contrast, the Freundlich model assumes that adsorption occurs on a heterogeneous surface, where adsorption sites exhibit different energies.

Both the Langmuir and Freundlich models were fitted to the experimental data for Pb­(II) adsorption (Figure 3).

**Figure 3:**
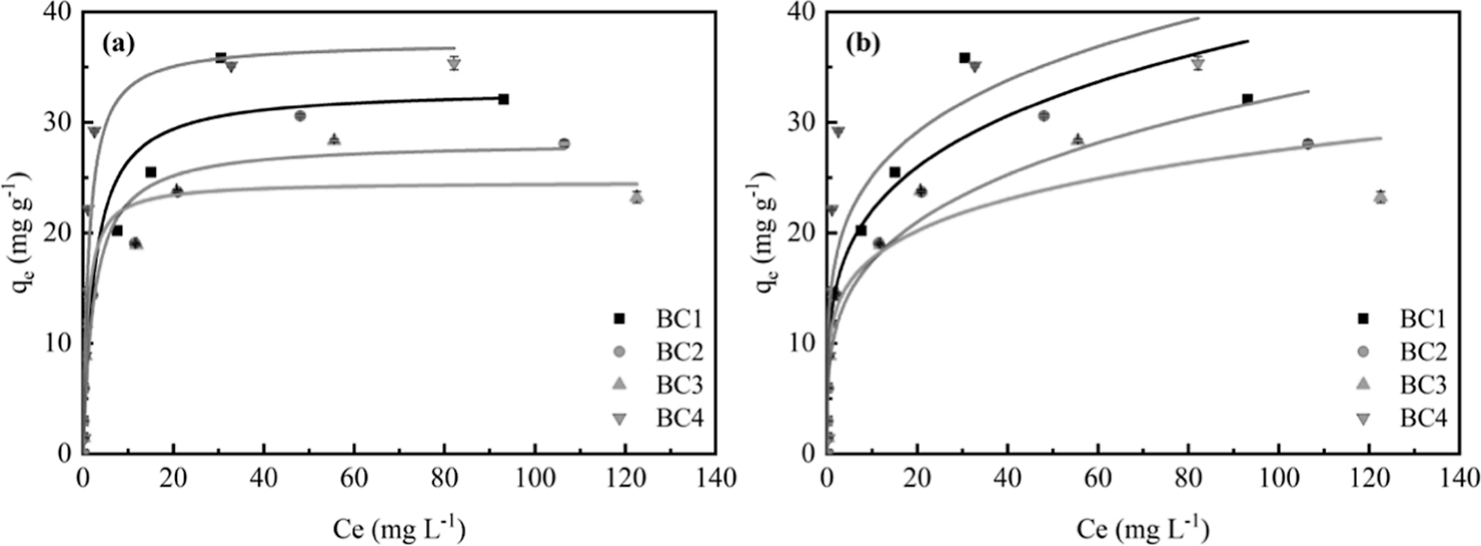
Pb­(II) adsorption data fitted to the (a) Langmuir and (b) Freundlich models. (Volume: 15 mL; biochar: 50 mg and size: 42 mesh; Pb­(II) concentration: 0–200 mg L^–1^; pH: 6.0; and contact time: 24 h).

The Pb­(II) adsorption isotherms with BC showed significant differences between the models, with the Langmuir monolayer isotherm better describing the removal of the Pb­(II) contaminant, considering all of the systems evaluated (Figure 3a, Table 1).

**Table 1:** Langmuir and Freundlich Parameters for the Isotherms of the Pb­(II) and Biochar (BC) System^a^

		Langmuir isotherm								Freundlich isotherm						
BC	*q*_exp_ (mg g^–1^)	*q*_max_ (mg g^–1^)	*K*_L_ (L mg^–1^)	*R*_L_	*R* ^2^	*R* ^2^-adjust	χ^2^	AIC	BIC	1/*n*	*K*_F_ (L mg^–1^)	*R* ^2^	*R* ^2^-adjust	χ^2^	AIC	BIC
BC1	35.84	33.06	0.4014	0.012	0.9385	0.9298	13.13	15.3	17.4	4.2301	12.7881	0.9349	0.9256	13.90	22.3	25.4
BC2	30.60	28.00	0.4483	0.011	0.9588	0.9529	6.68	15.4	17.5	3.8670	9.7748	0.9340	0.9246	10.68	22.2	25.3
BC3	28.34	24.62	0.9930	0.005	0.9516	0.9447	5.49	15.2	17.1	5.2274	11.3879	0.8723	0.8540	14.49	22.1	25.2
BC4	35.36	37.27	0.7916	0.006	0.9388	0.9301	12.86	15.5	17.6	4.6707	15.3467	0.7872	0.7569	44.73	22.4	25.5

a BC: biochar (BC1, 350 °C; BC2, 450 °C; BC3, 500 °C; BC4, 550 °C); *q*_exp_: experimental adsorption capacity; and *q*_max_: maximum adsorption capacity.

The Langmuir model provided a better fit to the experimental data, with *R* ^2^ values ≥0.9385 and adjusted *R* ^2^ ≥ 0.9298, in addition to lower χ^2^ values for all evaluated BC. These results are consistent with previous studies on BC for the removal of potentially toxic metals.^32–34^ According to this model, Pb­(II) adsorption on BC from CP occurs as a monolayer on a homogeneous surface, without interactions between adsorbed ions, and all surface sites have the same adsorption energy. Replicates of the adsorption studies showed variation <0.5%.

The dimensionless RL parameter describes the favorability of the adsorption. For an initial Pb­(II) concentration of 200 mg L^–1^, RL values between 0 and 1 indicate favorable adsorption.^35^

The Langmuir model showed lower AIC and BIC values compared to Freundlich, confirming its superior performance. The ΔAIC (>7) indicates strong evidence in favor of the Langmuir model.

Maximum adsorption capacity (*q*_max_) is a key parameter for evaluating the efficiency and feasibility of an adsorbent. Pyrolysis temperature influenced *q*_max_ for BC. According to the Langmuir model, *q*_max_ values for BC1, BC2, BC3, and BC4 were 33.06, 28.00, 24.62, and 37.27 mg g^–1^, respectively. The decrease in *q*_max_ from BC1 (350 °C) to BC3 (500 °C) of approximately 25.53% may be attributed to thermal degradation, reducing the functional groups available on the BC surface for interaction with Pb­(II).^36^ BC4 (550 °C) exhibited the highest *q*_max_, likely due to modifications in BC properties at higher pyrolysis temperatures, resulting in increased surface area and the presence of distinct functional groups, as confirmed by Thermogravimetric Analysis (TGA) and Fourier transform infrared spectroscopy (FTIR) data reported by Silva et al.^15^

The integrated analysis of the physicochemical properties of biochars and the adsorption results shows a direct relationship between the pyrolysis conditions and Pb­(II) removal performance. The decrease in H/C and O/C atomic ratios with increasing pyrolysis temperature indicates a higher degree of carbonization and aromaticity as well as a reduction in surface polarity. This behavior suggests the formation of more condensed and stable structures with a lower quantity of oxygenated functional groups, which may reduce the contribution of complexation-type interactions with surface groups. On the other hand, biochars produced at higher temperatures tend to have a higher ash content and a greater presence of mineral phases, which favors additional mechanisms, such as Pb­(II) precipitation (for example, as carbonates or hydroxides) and ion exchange with inorganic cations present in the matrix. High-temperature BC provides more adsorption sites, and both functional groups and mineral components contribute to the enhanced adsorption of Pb­(II).^36^

However, the results indicate that adsorptive performance is not governed exclusively by surface area but by a combination of factors, including elemental composition, presence of functional groups, and mineral content. In this context, biochar produced at 550 °C showed the best performance, which can be attributed to the balance between a more aromatic and stable structure, adequate porosity, and the presence of mineral components capable of promoting multiple Pb­(II) retention mechanisms. Thus, the results reinforce that Pb­(II) adsorption in biochar is a multifactorial process in which distinct physicochemical properties act synergistically, controlling both the capacity and the mechanism of contaminant removal.

Comparing the Pb­(II) adsorption capacities with the adsorption capacities published in the literature from different BC sources, a similarity between the results can be observed, especially when comparing the same pyrolysis temperature for the BC obtained (Table 2).

**Table 2:** Adsorption Capacity (*Q*_max_) for Different Biochar Sources (BC) and Pyrolysis Temperature Compared with Biochar (BC) from Cassava Peel (CP) and Pb­(II)

			isothermal model		
			Pb(II)		
biochar source	temperature (°C)	pH	*q*_max_ (mg g^–1^)		reference
poplar sawdust	400	5.0	38.08	Langmuir	^36^
	500		45.64		
	600		51.91		
	700		62.68		
	800		58.85		
rice husk	350–450	6.0	17.57	Langmuir	^37^
sawdust	450–550		14.20		
wheat straw pellets	700	-	113.64	Langmuir	^32^
rice husk		-	34.98		
softwood pellets	550	-	8.07		
cassava peel	350	6.0	33.06	Langmuir	this work
	450		28.00		
	500		24.62		
	550		37.27		

The differences in metal ion removal can be attributed to the specific properties of the BC and their affinities for the adsorption sites.^38^ Additionally, the raw material used for BC production affects the composition of chemical species present in the BC, which in turn influences the adsorption process, including potential interactions between coexisting ions.^39^

### 3.4 Equilibrium Time and Complexation Capacity

The complexation capacity (CC) study was conducted to evaluate the affinity of BC produced from CP at different pyrolysis temperatures for the Pb­(II) ions. CC represents the maximum amount of a given metal species, such as free Pb­(II), that can be complexed by BC in aqueous solution, as determined using the TF-UF technique.^21^

Given the similar behavior observed in the equilibrium time and CC curves for Pb­(II), the equilibrium time was illustrated using BC produced at the highest pyrolysis temperature (550 °C), while the CC was determined from the intersection of two linear segments using BC produced at the lowest temperature (350 °C), reflecting the interaction of BC with Pb­(II) (Figure 4, Table 3).^21^

**Figure 4:**
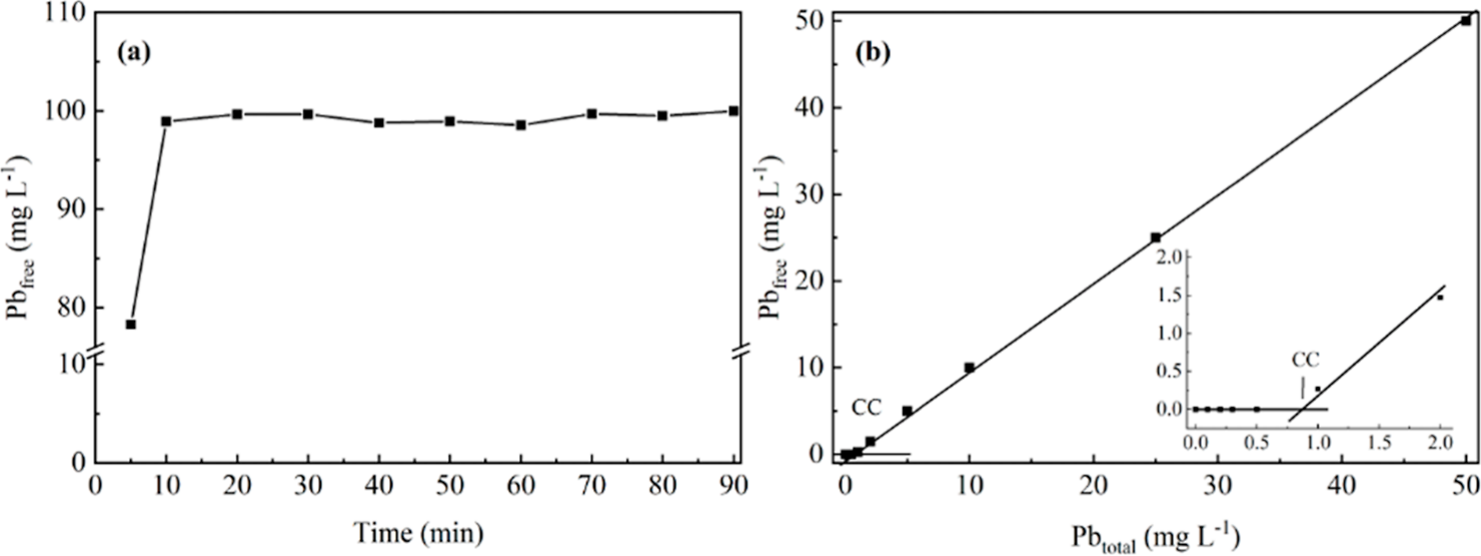
(a) Equilibrium time between lead and biochar produced from cassava peel. (b) Determination of complexation capacity using the UF-FT technique (Volume: 500 mL; biochar 10 mg and size: 42 mesh; Pb­(II) concentration for equilibrium study: 100 mg L^–1^; and Pb­(II) concentration for CC study: 0–50 mg L^–1^; pH 6.0).

**Table 3:** Complexation Capacity for Different Species Compared with Biochars Produced from Cassava Peel under Different Pyrolysis Temperatures (Volume: 500 mL; Biochar 10 mg and Size: 42 Mesh; Pb­(II) Concentration for CC Study: 0–50 mg L^–1^; and pH 6.0)^a^

material	metals	origin	CC (mg g^–1^)	reference
HS	Cu(II)	water	16.21	^42^
HS	Pb(II)	Peat	17.26	^21^
	Ca(II)		1.70	
	Mg(II)		1.05	
	Cu(II)		8.74	
	Al(III)		3.67	
BC1	Pb(II)	cassava peel	39.11	this work
BC2			38.14	
BC3			37.60	
BC4			38.39	

a BC: biochar (BC1, 350 °C; BC2, 450 °C; BC3, 500 °C; BC4, 550 °C); CC: complexation capacity; and HS: humic substances.

Complexation equilibrium was reached after approximately 10 min for all systems evaluated (Figure 5a). Pyrolysis temperature did not affect the equilibrium time. BC-Pb­(II) complexes stabilize over time as interactions with the ions intensify. This process involves intermolecular and/or intramolecular rearrangements, as well as the migration of complexed species to deeper sites within the BC structure.^23^

**Figure 5:**
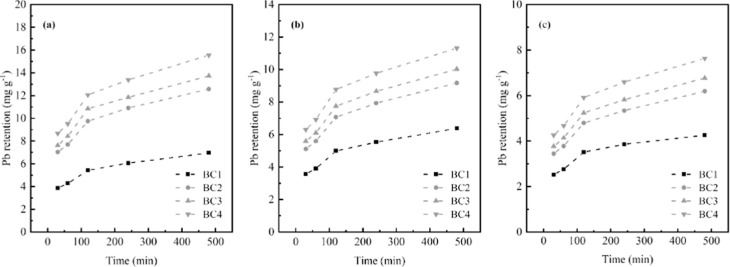
Pb­(II) retention as a function of in situ study time in the Mundaú-Manguaba estuarine lagoon complex, using the following masses: (a) 200 mg; (b) 300 mg; and (c) 500 mg, varying the contact time from 30 to 480 min.

The CC of natural materials, including humic substances, humic acids, humin, and BC, is an important parameter for understanding their interactions with contaminants and nutrients. [20,.^40^ CC is influenced by factors such as adsorbent concentration, concentration of the species to be complexed, pH, temperature, particle size, and other conditions.^21,41^ Therefore, CC values for BC produced at different pyrolysis temperatures are critical for assessing the quantity of contaminants that can be complexed and the material’s affinity for them. In all systems evaluated, BC exhibited the highest affinity for Pb­(II) (Table 3).

Pyrolysis temperature had little effect on the CC of Pb­(II). The BC produced at the lowest pyrolysis temperature (350 °C) exhibited the highest CC (39.11 mg g^–1^). This higher complexation capacity may be associated with the greater abundance of functional groups on the BC surface, as confirmed by the elemental analysis (Figure 2).

Several studies in the literature have employed soil humic substances (HS) as a source of organic matter for CC determination (Table 3). Botero et al.,^21^ reported CC values for HS from peat of 17.26 mg g^–1^ for Pb­(II), 1.70 mg g^–1^ for Ca­(II), 1.05 mg g^–1^ for Mg­(II), 8.74 mg g^–1^ for Cu­(II), and 3.67 mg g^–1^ for Al­(III). Rosa et al. (2006) obtained a CC of 16.21 mg g^–1^ for Cu­(II) in aquatic HS. When comparing Pb­(II), the CC values determined in this study were higher than those reported by Botero et al.,^21^ for HS from peat. This comparison suggests that Pb­(II) ions exhibit a particularly high CC with BC from CP.

### 3.5 In Situ Application of Biochars for Lead Retention

For the in situ application, the retention of the Pb­(II) contaminant was evaluated as a function of the variation in the mass of the BC used (Figure 5).

Increasing the BC mass used in the adsorption studies did not result in a greater retention of metal species across the evaluated systems (Figure 5). The highest Pb­(II) retention was observed with 200 mg of BC (Figure 5a), with retention values of approximately 8.67 mg g^–1^, 9.52 mg g^–1^, 12.07 mg g^–1^, and 15.56 mg g^–1^, respectively.

The environmental implications of biochar application in aquatic systems must be carefully considered, particularly under real conditions, such as those observed in lagoon Mundaú-Manguaba. Although biochar is widely recognized as an effective and low-cost adsorbent, its interaction with natural water may lead to secondary effects that influence overall water chemistry.

One relevant aspect is the potential release of mineral components inherent in the biochar matrix. Depending on the feedstock and pyrolysis conditions, biochars may contain ash fractions rich in alkali and alkaline earth metals (e.g., Ca, Mg, and K), as well as trace elements. When introduced into aqueous environments, especially under varying ionic strength and redox conditions, these components can be partially released, leading to changes in parameters, such as conductivity, alkalinity, and ionic composition. In lagoon systems, this process may alter nutrient balance and, in some cases, contribute to unintended shifts in the water quality.

The CC results (Table 3) and adsorption isotherm data (Table 1) confirmed the high affinity of Pb­(II) for BC, consistent with the in situ results (Figure 5). According to the Pb­(II) adsorption isotherms (Figure 3) and Langmuir parameters (Table 1), BC4 exhibited the highest *q*_max_ (37.27 mg g^–1^) and the highest Pb­(II) retention in the in situ study (15.56 mg g^–1^), demonstrating that the pyrolysis temperature influenced contaminant removal.

BC produced at higher pyrolysis temperatures generally develops specific functional groups and larger surface areas, enabling greater Pb­(II) adsorption than BC produced at lower temperatures.^15,36^ Moreover, the lower Pb­(II) adsorption observed in the in situ study compared to the CC results (Table 3) may be attributed to competition among different metal species in the environment for the available adsorption sites on BC.

Regarding retention time, Pb­(II) adsorption by BC under in situ conditions at MMELC reached its maximum at 480 min regardless of BC mass. This finding suggests that longer contact times under ambient conditions favor greater adsorption of the metal species. Therefore, BC4, produced at 550 °C and applied at a mass of 200 mg with a retention time of 480 min, exhibited the most effective conditions for Pb­(II) retention under ambient conditions.

We also highlight that the presence of other metallic ions in the lagoon water sample may compete with the removal process of Pb^2+^ ions. In this study, the water sample has the following levels: Ca 2.15 mg L^–1^, Fe 1.03 mg L^–1^, Mn 0.06 mg L^–1^, Al 0.05 mg L^–1^, Mg 0.87 mg L^–1^, and Cd < 0.01 mg L-1. In this initial evaluation, we observed the behavior of biochar in this complex matrix (lagoon water) and its potential as an environmental remediator.

When the results obtained in adsorption studies are compared with the retention results evaluated in in situ experiments, important differences are observed. While adsorption isotherms indicate high maximum capacities (*q*_max_) in controlled systems, these values reflect idealized conditions generally involving single-element solutions, constant pH, and the absence of interferents. On the other hand, in the natural lagoon environment, Pb­(II) retention was significantly lower, which can be attributed to the greater complexity of the medium, including competition with other dissolved cations (such as Ca^2+^ and Mg^2+^), the presence of natural organic matter, pH variations, and hydrodynamic conditions. Furthermore, processes such as pore blockage and coating of the biochar surface by organic and inorganic constituents can reduce the availability of active sites.

The promising results of applying biochar in a real-world system should be accompanied by further studies seeking to correlate important data from the evaluated environmental system. Due to the inherent complexity of the matrix, multiple factors such as organic matter, ionic strength, and hydrodynamic conditions, which influence the process, should be investigated. Thus, the in situ results represent a more realistic estimate of the material’s performance under environmental conditions, highlighting the importance of considering these limitations when extrapolating laboratory data to field applications.

## 4 Conclusions

The evaluation of BC produced from CP under different pyrolysis temperatures revealed its strong potential for the remediation of toxic metals in real environments. The most effective conditions were achieved at 550 °C, using 200 mg of BC and a retention time of 480 min. Under these conditions, the maximum lead adsorption capacity was 37.27 mg g^–1^, the complexation capacity reached 38.39 mg g^–1^, and the in situ retention was 15.56 mg g^–1^. Among the isotherm models, the Langmuir model showed the best fit to the experimental data, indicating monolayer adsorption on a homogeneous surface with no interaction between adsorbed ions.

Elemental analysis confirmed that increasing the pyrolysis temperature decreased H and O contents while increasing C and N contents. The resulting decreases in H/C, O/C, and N/C molar ratios reflect the degradation of functional groups and the formation of more stable aromatic ring structures. These structural changes make BC less susceptible to microbial degradation, thereby enhancing its persistence and effectiveness in environmental applications.

The economic viability of biochar derived from cassava waste stands out due to its low cost and wide availability in Brazil; however, large-scale implementation requires further studies for a more in-depth technical and economic evaluation.
